# Phage display assisted discovery of a pH‐dependent anti‐α‐cobratoxin antibody from a natural variable domain library

**DOI:** 10.1002/pro.4821

**Published:** 2023-12-01

**Authors:** Tulika Tulika, Rasmus W. Pedersen, Charlotte Rimbault, Shirin Ahmadi, Esperanza Rivera‐de‐Torre, Monica L. Fernández‐Quintero, Johannes R. Loeffler, Markus‐Frederik Bohn, Anne Ljungars, Line Ledsgaard, Bjørn G. Voldborg, Fulgencio Ruso‐Julve, Jan Terje Andersen, Andreas H. Laustsen

**Affiliations:** ^1^ Department of Biotechnology and Biomedicine Technical University of Denmark Lyngby Denmark; ^2^ Center for Molecular Biosciences Innsbruck, Department of General Inorganic and Theoretical Chemistry, University of Innsbruck Innsbruck Austria; ^3^ Department of Pharmacology University of Oslo Oslo Norway; ^4^ Department of Immunology Oslo University Hospital Rikshospitalet Oslo Norway; ^5^ Precision Immunotherapy Alliance University of Oslo Oslo Norway

**Keywords:** acid‐switched antibodies, antibody developability, antibody recycling, chain‐shuffling, pH‐dependent antibodies, phage display technology, snake venom, snake toxins

## Abstract

Recycling IgG antibodies bind to their target antigen at physiological pH in the blood stream and release them upon endocytosis when pH levels drop, allowing the IgG antibodies to be recycled into circulation via FcRn‐mediated cellular pathways, while the antigens undergo lysosomal degradation. This enables recycling antibodies to achieve comparable therapeutic effect at lower doses than their non‐recycling counterparts. The development of such antibodies is typically achieved by histidine doping of their variable regions or by performing *in vitro* antibody selection campaigns utilizing histidine doped libraries. Both are strategies that may introduce sequence liabilities. Here, we present a methodology that employs a naïve antibody phage display library, consisting of natural variable domains, to discover antibodies that bind α‐cobratoxin from the venom of *Naja kaouthia* in a pH‐dependent manner. As a result, an antibody was discovered that exhibits a 7‐fold higher off‐rate at pH 5.5 than pH 7.4 in bio‐layer interferometry experiments. Interestingly, no histidine residues were found in its variable domains, and in addition, the antibody showed pH‐dependent binding to a histidine‐devoid antigen mutant. As such, the results demonstrate that pH‐dependent antigen‐antibody binding may not always be driven by histidine residues. By employing molecular dynamics simulations, different protonation states of titratable residues were found, which potentially could be responsible for the observed pH‐dependent antigen binding properties of the antibody. Finally, given the typically high diversity of naïve antibody libraries, the methodology presented here can likely be applied to discover recycling antibodies against different targets *ab initio* without the need for histidine doping.

## 1 INTRODUCTION

Monoclonal antibodies (mAbs) of the immunoglobulin G (IgG) class are a rapidly growing class of drugs (Kaplon et al., 2022) used to treat a range of conditions, including cancer and autoimmune diseases (Carter & Lazar, 2018; Carter & Rajpal, 2022). Two of the major factors behind their clinical success include their high specificity and affinity for their cognate antigens combined with the ability to mediate effector functions (Carter & Lazar, 2018; Carter & Rajpal, 2022). In addition, IgGs have a plasma half‐life of 3 weeks on average in humans, which makes them an attractive choice for the development of mAbs for diseases where exposure over time is key. This hallmark is regulated by binding of the IgG fragment crystallizable (Fc) region to a broadly expressed cellular receptor named the neonatal Fc receptor (FcRn), which rescues IgGs from intracellular lysosomal degradation via recycling or transcytosis. Mechanistically, this happens in a strictly pH‐dependent manner where IgGs enter cells via fluid‐phase pinocytosis followed by engagement of FcRn, which predominantly resides in mildly acidified endosomes. The complexes are then recycled back to the cell surface or transcytosed across polarized cells followed by exposure to the near neutral pH, which triggers dissociation and release of the IgGs to the extracellular milieu (Challa et al., 2014; Lencer & Blumberg, 2005). Thus, IgGs are rescued from intracellular degradation via FcRn‐directed transport routes.

However, when most IgGs are bound to their cognate antigens, this often occurs with high affinity throughout the endosomal pH gradient. IgGs may therefore undergo antigen‐mediated clearance via lysosomal degradation or they may be recycled by FcRn along with the bound antigen. As a result, each antibody paratope can clear an antigen only once in their lifetime. To enhance this ability, an attractive strategy is to engineer the binding properties of the antibody such that high affinity to the antigen is kept at near neutral pH, while binding affinity becomes weaker when the antibody–antigen complex enters the acidic environment of endosomes (Bonvin et al., 2015; Chaparro‐Riggers et al., 2012; Devanaboyina et al., 2013; Igawa et al., 2010; Igawa et al., 2013; Igawa et al., 2016; Klaus & Deshmukh, 2021; Lee et al., 2019; Schröter et al., 2015; Sheridan et al., 2018). This allows the antigen to dissociate from the antibody in the acidic endosomes (pH 5.0–6.5) and be transported to the lysosomes for degradation, while the antibody is rescued via FcRn‐mediated pathways and released upon exposure to the near‐neutral pH conditions (pH~7.4) at the cell surface. This allows the same IgG molecule to clear antigens multiple times in the blood stream (Igawa et al., 2013; Klaus & Deshmukh, 2021; Roopenian & Akilesh, 2007) and is here defined as a “recycling antibody”. Such engineering has been shown to reduce the required dose and/or frequency of dosing to achieve therapeutic effect (Fukuzawa et al., 2017; Lee et al., 2019). Importantly, this could also be an attractive approach for the design of antibodies tailored for treatment regimens relying on high dosing and where cost is a limiting factor, such as snakebite envenoming and infectious diseases (Laustsen, 2019).

Specifically, the ability of antibodies to bind their cognate antigens in a pH‐dependent manner has largely been attributed to the presence of histidine residues (p*K*_*a*_ ~ 6.0) at the antibody–antigen binding interface (Raghavan et al., 1995; Tanokura, 1983). Thus, the discovery of antibodies with pH‐dependent antigen binding properties has predominantly been carried out using histidine scanning approaches or histidine‐enriched libraries (Bonvin et al., 2015; Chaparro‐Riggers et al., 2012; Devanaboyina et al., 2013; Igawa et al., 2010; Igawa et al., 2014; Ito et al., 1992; Könning et al., 2016; Laughlin & Horn, 2022; Murtaugh et al., 2011; Schröter et al., 2015; Watkins & Watkins, 2022). However, such strategies may not always be straightforward, as histidine doping may compromise target binding properties at neutral pH (Devanaboyina et al., 2013; Igawa et al., 2014; Laughlin & Horn, 2022). In addition, histidine‐mediated pH‐dependent binding requires the epitope to have positively charged residues, which restricts the number of suitable epitopes (Ledsgaard, Ljungars, et al., 2022). Finally, histidine‐enriched antibody libraries are generated synthetically, and therefore, the resulting antibodies present in these libraries may have developability and immunogenicity risks that should be taken into consideration. Immune libraries derived from animals have also been explored, but antibodies derived from these have needed to undergo humanization, which presents further complications for antibody development (Sampei et al., 2018; Yang et al., 2017). Additional approaches for the discovery of antibodies with pH‐dependent antigen binding properties are thus attractive.

In this study, we demonstrate the utility of natural naïve human antibody libraries for the discovery of fully human IgG1 antibodies with pH‐dependent antigen binding properties. We show that this is possible even without having any histidine residues in the complementarity‐determining regions (CDRs). To achieve this, we employed phage display technology and a naïve human antibody library consisting of naturally occurring variable domains for the discovery of antibodies with pH‐dependent binding properties against a three‐finger toxin (3FTx), specifically the long‐chain α‐neurotoxin, α‐cobratoxin (α‐cbtx). We also demonstrated that the single histidine residue present in position 18 of α‐cbtx does not contribute to the pH‐dependence of the antibody–antigen binding interaction. Finally, we employed molecular dynamics simulations to structurally characterize potential factors and residues responsible for the pH‐dependency of the discovered antibody, highlighting the importance of protonation states on antibody structure and dynamics. The results showcase that pH‐dependency of antibody–antigen binding interactions is not always driven by histidine residues, but instead can be due to other structural features at the binding interface.

## 2 RESULTS

### 2.1 pH elution during antibody phage display selections enables discovery of binders with pH‐dependent antigen binding properties

**FIGURE 1:**
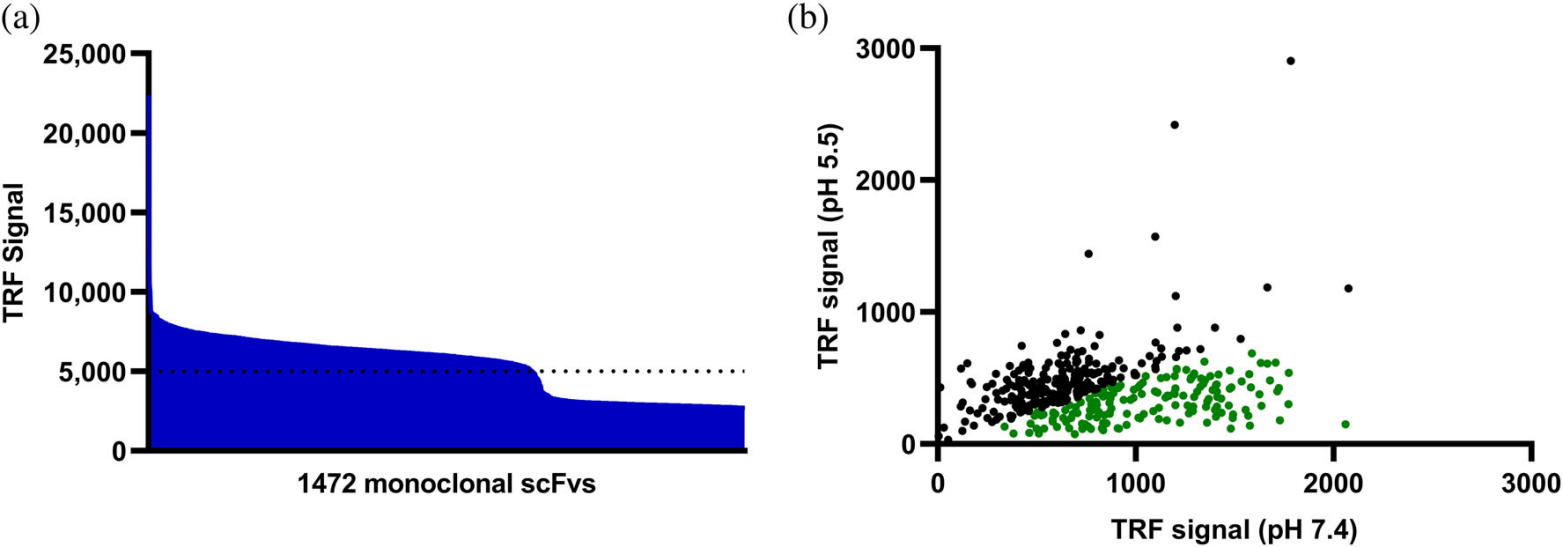
Screening of binders. (a) Binding signal as Time‐Resolved Fluorescence (TRF) of 1472 discovered monoclonal single‐chain variable fragments (scFvs) against α‐cobratoxin (α‐cbtx) in expression‐normalized capture (ENC) dissociation‐enhanced lanthanoid‐based fluorescence assay (DELFIA). (b) Scatter plot showing binding signal as TRF of a subset of selected α‐cbtx binding monoclonal scFvs in ENC pH DELFIA. The monoclonal scFvs showing at least 50% decrease in binding signal after being incubated in a buffer at pH 5.5 compared to pH 7.4 are colored in green.

To enrich antibodies with pH‐dependent antigen binding properties, three consecutive rounds of phage display selections were performed against biotinylated α‐cbtx using a buffer with low pH or trypsin (as a control) for elution of binding single‐chain variable fragment (scFv) displayed on phages. Following reformatting to soluble scFv and expression in *Escherichia coli*, 918 of the 1472 screened clones bound to α‐cbtx in an expression‐normalized capture (ENC) dissociation‐enhanced lanthanide fluorescence immunoassay (DELFIA) (Laustsen et al., 2018; Martin et al., 2006) with a 5 times higher signal than the negative control giving a cut‐off value of 5000 (Figure 1A). To screen for binders with pH‐dependent antigen binding properties, 635 of the binding clones were randomly selected, re‐expressed, and analyzed in an ENC pH DELFIA, where the clones were allowed to bind α‐cbtx at pH 7.4 and thereafter either incubated in a buffer of pH 7.4 or pH 5.4 for an h before adding the detection reagent. This revealed that 166 clones showed at least 50% decrease in the binding signal after incubation at pH 5.5 compared to 7.4, indicating a pH‐dependent binding of the scFvs to the antigen (Figure 1B; Bonvin et al., 2015). Further, sequencing of these 166 clones showed that ~99% of the clones were identical, resulting in 2 unique clones. Both clones came from the phage display selection where a low pH buffer was employed for elution of the bound phages.

### 2.2 The most abundant clone with pH‐dependent antigen binding properties contains no histidine residues in the variable domains

**FIGURE 2:**
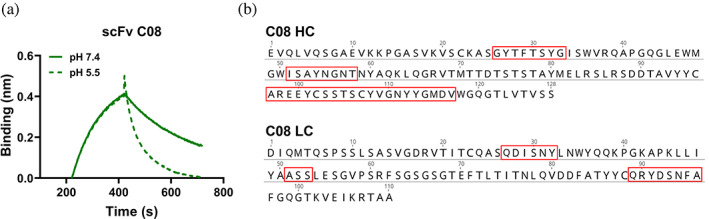
Bio‐layer interferometry (BLI) binding curves and sequence of the clone C08 with pH‐dependent antigen binding properties. (a) BLI sensogram showing association at pH 7.4 and dissociation at pH 7.4 (solid line) and pH 5.5 (dashed line) for scFv C08. (b) Amino acid sequences of heavy chain (HC) and light chain (LC) variable domain of clone C08. IMGT numbering was used and the CDRs are highlighted in red boxes.

The two unique scFv clones were expressed in *E. coli*, and the bacterial supernatants containing the expressed scFvs were used in bio‐layer interferometry (BLI) experiments to determine their pH‐dependent target dissociation. The most abundant clone, TPL0197_01_C08 (which will be referred to as C08 from here on) showed a faster dissociation at pH 5.5 compared to pH 7.4 (Figure 2A). Sequence analysis of this clone showed that it contains no histidine residues in the variable regions‐ neither in the CDRs nor in the framework (Figure 2B). This was surprising since histidine residues have been widely attributed as a major contributing factor of pH‐dependent binding (Klaus & Deshmukh, 2021).

To investigate the antibody specificity, the binding of C08 towards several other toxins and the binding in presence of human serum were assessed using BLI. To avoid potential avidity affects in the analysis, C08 was reformatted to a Fab format, expressed in CHO cells, and purified on Ni‐NTA using the His‐tag. The toxins included in the binding analysis were a long‐chain α‐neurotoxins (LNTx) from *Bungarus multicinctus*, which is structurally similar to α‐cbtx, a short‐chain α‐neurotoxin (SNTx) from *Micrurus diastema*, and a phospholipase A_2_ (PLA_2_) from *Micrurus fulvius*. This analysis revealed that Fab C08 bound to α‐cbtx but not to any of the other toxins (Figure S1). Thereafter the binding of Fab C08 to α‐cbtx in the presence of 10 or 50% human serum were assessed, which revealed that Fab C08 binds to α‐cbtx also in the presence of human serum. Combined, this demonstrates the specificity of C08 for α‐cbtx (Figure S1).

### 2.3 Fab C08 with pH‐dependent antigen binding properties shows an increased rate of dissociation at pH 5.5

**FIGURE 3:**
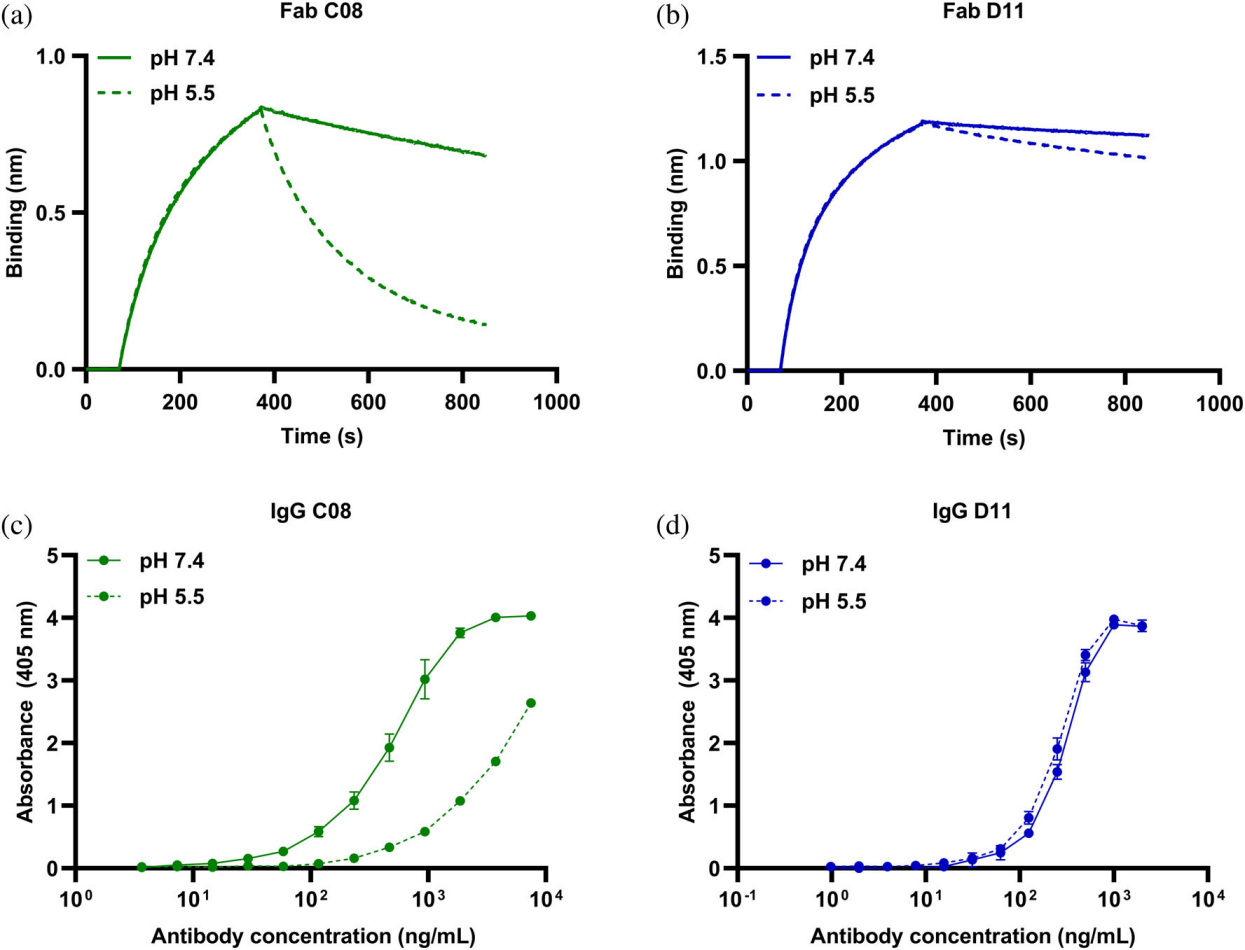
Binding characterization of a clone with pH‐dependent (C08) and a clone with non‐pH‐dependent antigen binding properties (D11) in Fab and IgG formats. Bio‐layer interferometry (BLI) curves showing association at pH 7.4 and dissociation at pH 7.4 (solid line) and 5.5 (dashed line) for α‐cobratoxin (α‐cbtx) binding (a) Fab C08 with pH‐dependent antigen binding properties at 300 nM and (b) Fab D11 without pH‐dependent antigen binding properties at 240 nM. ELISA binding curves to α‐cbtx at pH 7.4 (solid line) and pH 5.5 (dashed line) of (c) IgG C08 with pH‐dependent antigen binding properties and (d) IgG D11 without pH‐dependent antigen binding properties.

To determine the binding kinetics while avoiding avidity affects, the Fab format of C08 was used. An α‐cbtx targeting Fab 2554_01_D11 (which will be referred to as D11 in the following text) that was previously discovered through phage display without any pH‐selection pressure, was included for comparison (Ledsgaard et al., 2023). Both Fabs were then characterized for their affinities at neutral pH and their pH‐dependent dissociation at neutral and low pH using BLI. The mean affinities of Fab C08 and Fab D11 at pH 7.4 was determined to be 44.8 nM and 4.7 nM respectively (Table 1). Next, to determine their pH‐dependent antigen binding characteristics, the Fabs were allowed to associate at pH 7.4 followed by dissociation at either pH 7.4 or 5.5 (Figure 3A,B). A 7‐fold higher off‐rate at pH 5.5 than 7.4 was observed for Fab C08. In comparison, the difference between the off‐rates at these pH values was 1.8‐fold for Fab D11 (Table 1).

**TABLE 1:** Kinetic rate constants and T_M_ of anti‐α‐cbtx Fabs.

Fab	K_D_ (nM)	*k*_off_ (10^−4^·s^−1^)	*k*_off_ (10^−4^·s^−1^)	*k*_off_ fold difference	Mean T_m_ (°C)	
	pH 7.4	pH 7.4	pH 5.5	(pH 5.5/7.4)	pH 7.4	pH 5.5
C08	44.8 ± 1.7	7.6 ± 0.14	53.5 ± 2.2	7.0	72.8 ± 0.01	73.1 ± 0.07
D11	4.7 ± 0.45	2.0 ± 0.05	3.7 ± 0.23	1.8	77.8 ± 0.08	78.6 ± 0.06

*Note*: Mean affinities at pH 7.4, off‐rates, and T_M_ of the Fab C08 with pH‐dependent antigen binding properties and the Fab D11 without pH‐dependent antigen binding properties at pH 7.4 and 5.5. The data represents the mean values ± SD, *n* = 3.

Further, to confirm that the increase in dissociation of C08 from α‐cbtx at pH 5.5 compared to pH 7.4 was not due to denaturation of the antibody at low pH, the melting temperatures (T_m_) for Fab C08 and Fab D11 were determined at pH 7.4 and pH 5.5 using Nano Differential Scanning Fluorimetry (NanoDSF). The obtained T_m_ values of the Fabs at neutral and acidic conditions were very similar, suggesting that the pH‐dependent antigen binding property of Fab C08 was not due to reduced stability at a low pH (Table 1).

### 2.4 IgG1 C08 with pH‐dependent antigen binding properties shows less binding at pH 5.5 compared to pH 7.4 in ELISA

To further characterize C08, it was reformatted to an IgG1 format, expressed in CHO cells, and purified. To validate the pH‐dependent binding between IgG1 C08 and α‐cbtx, an ELISA‐based binding assay was performed at pH 7.4 and 5.5. D11 in IgG1 format was included as a control for exhibiting non‐pH‐dependent antigen binding properties. Both IgG1s bound to α‐cbtx at pH 7.4, however the signal at comparative concentrations was lower for C08 than D11, indicating a lower affinity of C08 towards α‐cbtx (Figure 3C,D). At pH 5.5, C08 showed lower binding to α‐cbtx, while D11 bound with almost identical strength to α‐cbtx as that observed at pH 7.4 (Figure 3C,D). This showed that both binding and pH‐dependency of C08 towards α‐cbtx was retained after reformatting to an IgG1 format.

### 2.5 The pH‐dependent binding between Fab C08 and α‐cbtx is independent of histidine residues

**FIGURE 4:**
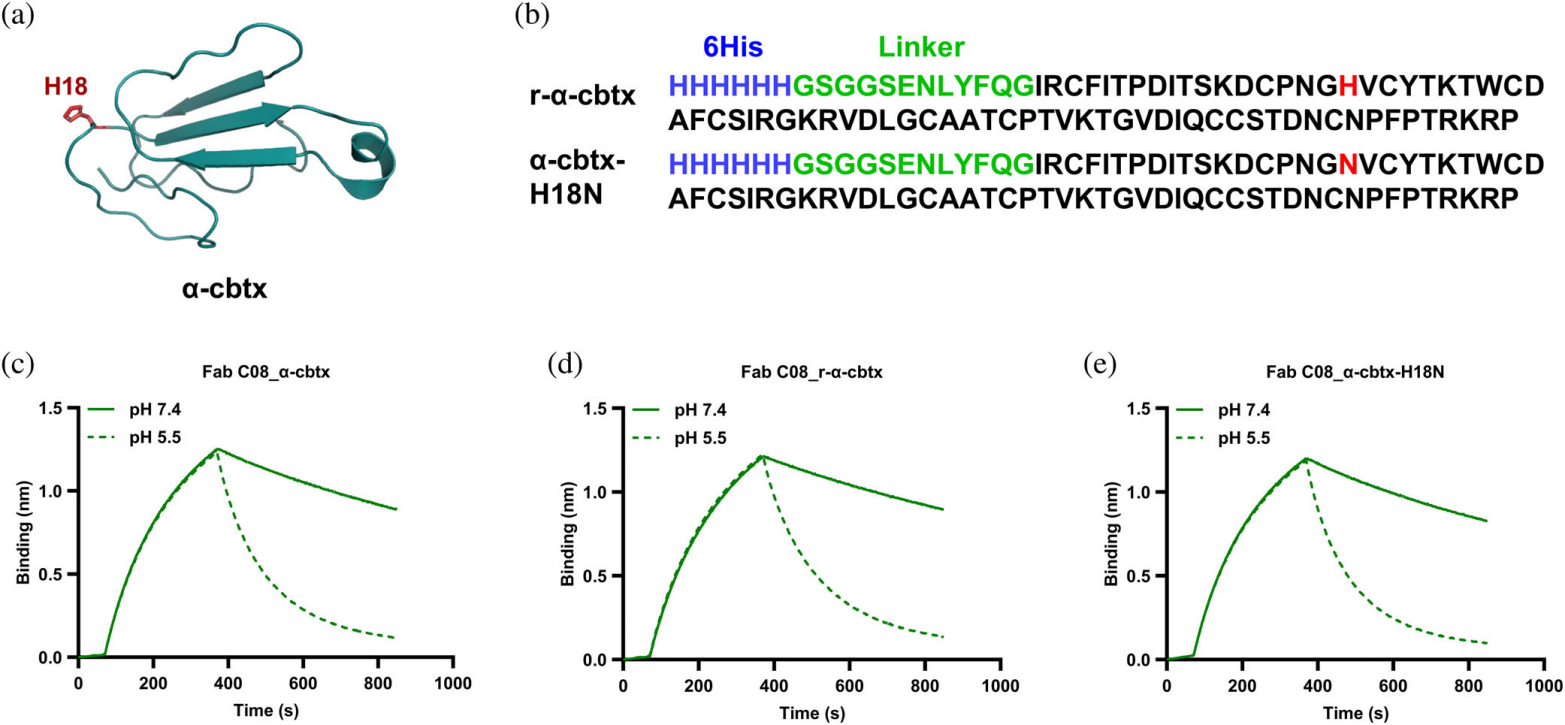
Native and recombinant α‐cobratoxin (α‐cbtx) and pH‐dependent binding of Fab C08 to the α‐cbtx variants. (a) Representation of a three‐dimensional crystal structure of α‐cbtx shown as cartoon in teal (PDB: 1CTX). The histidine residue present at position 18 in α‐cbtx is shown in red stick. (b) Amino acid sequences of recombinant wildtype α‐cbtx (r‐α‐cbtx) and mutant α‐cbtx‐H18N. The histidine residue (H) in α‐cbtx is mutated to an asparagine residue (N) in α‐cbtx‐H18N (marked in red). Both the r‐α‐cbtx and α‐cbtx‐H18N contain a 6xHis tag (blue) separated by a linker (green) at the N‐terminal of the toxin. (c) Bio‐layer interferometry (BLI) curves showing association at pH 7.4 and dissociation at pH 7.4 (solid line) and pH 5.5 (dashed line) of Fab C08 at 300 nM from native α‐cbtx, (d) r‐α‐cbtx, and (e) α‐cbtx‐H18N.

Although the variable region of the C08 is devoid of histidine residues, its cognate antigen, α‐cbtx, contains one histidine residue at position 18 (Figure 4A). To investigate whether the observed pH‐dependent binding between C08 and α‐cbtx derived from the histidine residue present in the antigen, a mutant α‐cbtx where the histidine in position 18 was replaced by an asparagine (α‐cbtx‐H18N) was recombinantly expressed and produced in *Komagataella phaffii* (formerly known as *Pichia pastoris*) and purified on Ni‐NTA using the His‐Tag (Figure S2). A recombinant wildtype α‐cbtx (r‐α‐cbtx) was produced as a control (Figure 4B). The secondary structures of the recombinant toxins were evaluated using circular dichroism (CD), which revealed a relative minimum ellipticity at 207 nm, indicating a high content of β‐sheet structure, matching the expected spectra of a 3FTx archetypical fold (Hider et al., 1982) and suggesting that the recombinant toxins were correctly folded (Figure S2). The recombinant toxins were then biotinylated for binding experiments. The pH‐dependent binding of Fab C08 against the native α‐cbtx, r‐α‐cbtx, and α‐cbtx‐H18N was evaluated using BLI, where Fab C08 exhibited similar dissociation profiles from the three antigens at pH 5.5, showing that the pH‐dependent interaction between the C08 and α‐cbtx is independent of the histidine residue in position 18 of α‐cbtx (Figure 4C–E).

### 2.6 Increase in conformational entropy of C08 contributing to the loss in affinity at pH 5.5

**FIGURE 5:**
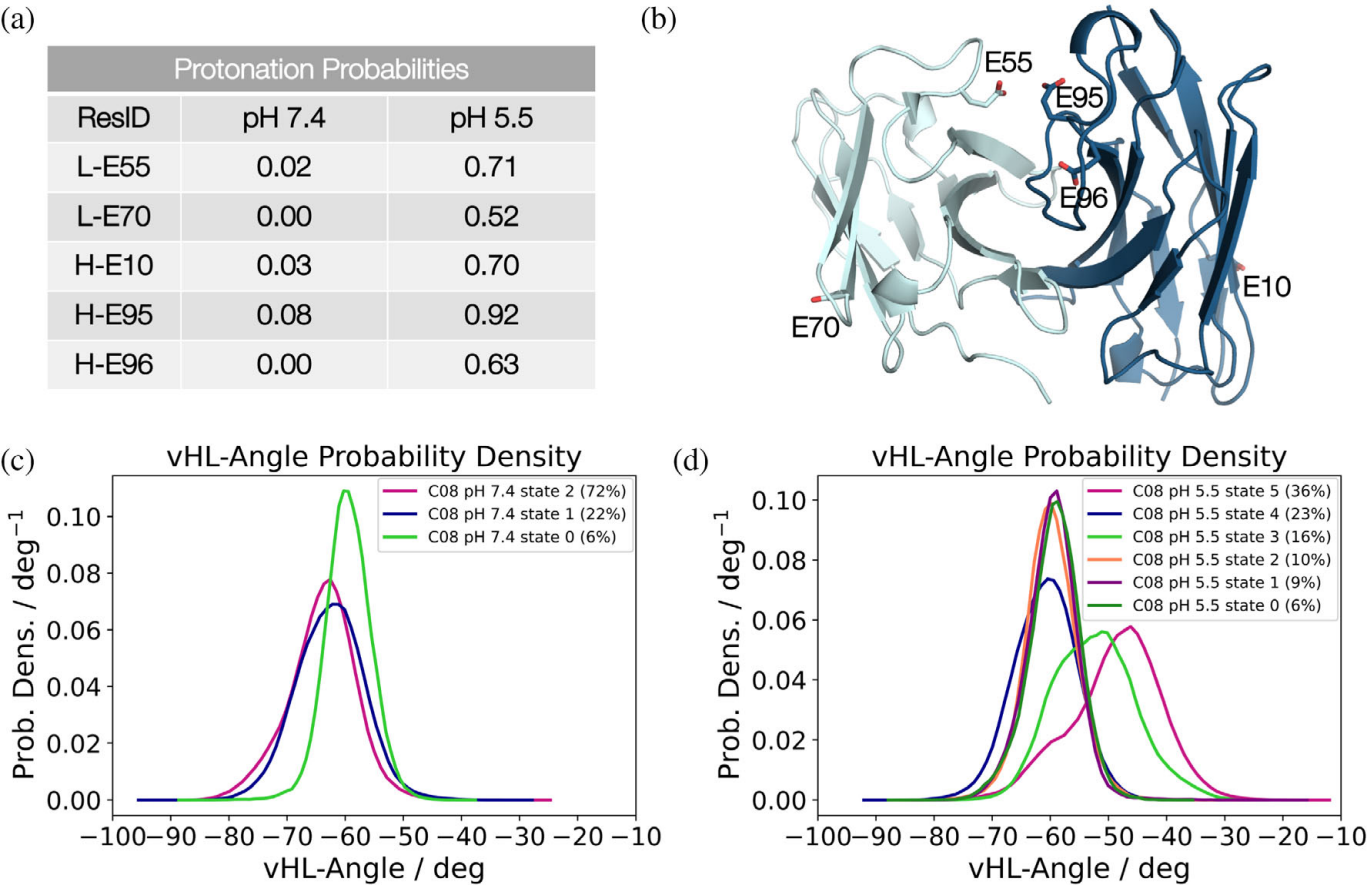
Role of different protonation states of specific residues on antibody dynamics. (a) Protonation probabilities for the residues in the heavy (H) and light (L) chain of antibody C08 that change protonation at pH 5.5. (b) Representative C08 variable fragment (Fv) structure showing the positions of the residues presented in (a). The heavy chain is colored in dark blue, and the light chain is depicted in cyan. Color‐coded relative V_H_‐V_L_ interdomain orientation (vHL‐Angle) distributions at (c) pH 7.4 and (d) pH 5.5 for the respective conformational states obtained from the Markov‐state model. The most probable state in solution of the free C08 antibody is shown in pink.

To mechanistically characterize the pH‐dependent functional changes in C08 at atomistic detail and to identify the critical residues potentially involved in sensing the changes in pH, molecular dynamics simulations were performed using the C08 variable region sequence at different protonation states. Thus, to calculate the most probable protonation states at pH 7.4 and pH 5.5 of the C08 in the absence of antigen, constant pH (cpH) molecular dynamics (MD) simulations were performed. The results of the cpH MD simulations are shown in Figure 5A. Three glutamate residues in the CDR‐H3 (E95, E96) and CDR‐L2 (E55) loops show a high probability to be protonated at pH 5.5, together with two outer solvent exposed framework residues (H‐E10, L‐E70) (Figure 5B). This is a strong indicator that these residues sense changes in pH first and therefore might be involved in co‐determining the pH‐dependent antigen binding properties of C08. To characterize structural changes at pH 7.4 and pH 5.5, the obtained protonation probabilities were used as input for molecular dynamics simulations to reconstruct thermodynamics and kinetics of protonation‐dependent conformational rearrangements using the Markov‐state model. Markov‐state models are used to reproduce long‐timescale statistical conformational dynamics and facilitate the thermodynamic and kinetic characterization of biomolecular mechanisms (Chodera & Noé, 2014). This resulted in three states for C08 at pH 7.4 and in six states for C08 at pH 5.5 from the Markov‐state model. Here, the observed increase in number of conformational states goes hand in hand with an increase in conformational entropy at low pH, which can have a detrimental effect on antigen binding. The overall increase in flexibility of the variable region of C08 is reflected in Figure 5C,D, showing a higher variability in the relative interdomain V_H_‐V_L_ orientation at pH 5.5. Additionally, the most probable states in solution at pH 7.4 and pH 5.5 reveal substantial shifts in the relative V_H_‐V_L_ orientation (colored in pink). In addition to the increase in conformational diversity and changes in the V_H_‐V_L_ orientation, different interaction networks originating from changes in protonation in the CDR loop residues (L‐E55, H‐E95, and H‐E96) at pH 5.5 compared to pH 7.4 were also found. Residues H‐E95 and H‐E96 form long‐lasting intramolecular hydrogen bonds (>80%) at pH 5.5, while at pH 7.4, the repulsion of these residues prohibits this interaction. In addition, the interaction probabilities of L‐E55 with L‐S56 decrease substantially from 61% at pH 7.4 to 37% at pH 5.5, as a consequence of the protonation. Thus, the different protonation states of the histidine‐free C08 do not only influence the conformational diversity of the variable region, but also result in distinct interaction networks and protonation‐dependent conformational states, highlighting the critical role that these residues may have in determining the pH‐dependent antigen binding properties of C08.

## 3 DISCUSSION

Recycling antibodies, that is, antibodies that engage with their target antigen in a pH‐dependent manner have gained increased attention, as they retain high efficacy when administered at lower doses than their counterparts without pH‐dependent antigen binding properties. This feature stems from the combination of pH‐dependent antigen binding properties and long plasma half‐life driven by FcRn‐mediated cellular recycling and transcytosis. Consequently, various strategies to discover and engineer antibodies with pH‐dependent antigen binding properties have been explored (Igawa et al., 2016).

In this study, we employed a naïve human antibody phage display library consisting of naturally occurring variable domains and successfully discovered an antibody, C08, that binds its target antigen in a pH‐dependent manner. While antibodies with pH‐dependent antigen binding properties have previously been discovered using histidine doping of antibody libraries or the paratopes of a pre‐existing antibody without pH‐dependent antigen binding properties (Igawa et al., 2016), we here show that such strategies may not necessarily be required to discover antibodies with highly pH‐dependent antigen binding properties from antibody libraries comprising native V_H_ and V_L_ domains. While previous studies have avoided histidine doping by utilizing immune libraries to isolate antibodies with pH‐dependent antigen binding properties comprising naturally occurring variable domains (Hinz et al., 2020; Yang et al., 2017), generation of such libraries may not always be feasible, and may prove to be challenging to construct depending on the toxicity and immunogenicity of the antigen.

Avoiding the incorporation of histidine residues in the variable regions of antibodies may have several benefits. First, introduction of such mutations in antibody paratopes can be a time‐consuming and laborious task, as it requires detailed analysis and validation of the pH‐dependent antigen binding properties of the mutants and may still not provide an antibody with desirable features. For example, it has been reported that, occasionally, histidine mutations that were incorporated to reduce the binding affinity between an antibody and its cognate antigen at low pH also resulted in reduced binding affinity at neutral pH (Devanaboyina et al., 2013; Igawa et al., 2016). Second, the incorporation of mutations, particularly in antibody sequences deriving from natural sources, may come with a risk of introducing sequence liabilities that could pose problems for the developability of the antibody, such as causing it to be immunogenic (Egli et al., 2022; Strohl, 2012; Ausserwöger et al., 2022; Fernández‐Quintero et al., 2023). The employment of an antibody library with natural variable domains for the discovery of leads with pH‐dependent antigen binding properties could potentially reduce this risk, since their variable domains have gone through the test for self‐tolerance (Schofield et al., 2007). Third, given that a naïve natural antibody library can be used to find binders to a multitude of targets, the observation that histidine doping may not be necessary for the discovery of antibodies with pH‐dependent antigen binding properties indicates that such antibodies can possibly be found against many targets directly *ab initio* (Laustsen et al., 2021).

Besides originating from a naïve natural antibody library, the variable domains of the discovered antibody with pH‐dependent antigen binding properties were surprisingly found to be entirely devoid of histidine residues. However, the native antigen (α‐cbtx) itself contains a histidine residue in position 18. We therefore investigated the binding interaction between the antibody C08 and a recombinantly expressed mutant of α‐cbtx, which lacked the histidine residue in position 18, and found that the binding interaction retained its pH‐dependence. This thus demonstrates that the pH‐dependent binding interaction between the antigen and the antibody did not involve the histidine residue at all. Although the presence of histidine residues is widely attributed to mediate pH‐dependent binding (Sampei et al., 2018), our findings thus suggest that antibodies can, at least at times, derive pH‐dependent antigen binding properties from different residues. A possible explanation for this could be the effect of the molecular microenvironment that can change the local p*K*_*a*_ of amino acid residues at the antibody–antigen binding interface, which may influence the binding behavior between the two molecules (Isom et al., 2011; Shan & Mehler, 2011).

To explore the influence of different protonation states on the structure and dynamics of C08 and to identify potential determinants for the pH‐dependent antigen binding properties of this antibody, we performed molecular dynamics simulations. The pH of the solution can have dramatic effects on the charge distribution of proteins, which in turn affects their fundamental structure and function. The dynamic properties of antibodies strongly depend on the pH‐dependent protonation states of the titratable residues since they govern the functionally relevant inter‐ and intramolecular interaction networks. Thus, characterizing the consequences of changes in protonation on the respective conformational ensembles of the variable antibody regions in solution can provide mechanistic insights in identifying determinants for pH‐dependent antibody–antigen recognition. Our simulations of the C08 antibody at pH 7.4 and pH 5.5 indicated that a substantially higher conformational diversity existed for this antibody at pH 5.5, which is reflected by the presence of more diverse conformational states, changes in the interaction networks, and a higher variability in the relative V_H_‐V_L_ interdomain orientation. This means that not only the CDR loops, but also the relative V_H_‐V_L_ interdomain orientation are affected by changes in protonation, resulting in an increased flexibility. This increase in conformational entropy, in combination with the shifts in probability density for the V_H_‐V_L_ interdomain orientations of the most dominant states in solution, potentially contribute to the decreased antibody–antigen binding affinity at pH 5.5.

So far, the utility of recycling antibodies that bind their target antigens in a pH‐dependent manner has mostly been demonstrated against endogenous targets, such as IL‐6, PCK9, CXCL10, and TNF‐α (Bonvin et al., 2015; Chaparro‐Riggers et al., 2012; Igawa et al., 2010; Schröter et al., 2015). In this study, an exogenous soluble antigen, α‐cbtx from *N. kaouthia* venom, was used as the target antigen. Recycling antibodies that bind snake toxins in a pH‐dependent manner could potentially find utility for the development of novel types of antivenoms, which could be administered to patients at lower doses compared to both current plasma‐derived antivenoms and recombinant antivenoms based on non‐recycling antibodies. However, in the case of snakebite envenoming, both complex toxicokinetics and pharmacokinetics are at play (Sanhajariya et al., 2018). While endogenous targets are often (semi‐)constitutively produced within the body and therefore can be maintained at a concentration below certain thresholds by using recycling antibodies; toxins are instantaneously injected in a large dose into the body of the victim during a snakebite envenoming case, and thus, require urgent intervention (Laustsen, 2019; Sanhajariya et al., 2018). The effectiveness of recycling antibodies with pH‐dependent antigen binding properties under such circumstances, where large amounts of fast‐acting toxins are required to be removed from circulation is not known. However, given that, in the majority of the cases of snakebite envenoming, the bite is either intramuscular or subcutaneous, the injection of toxins is followed by an initial absorption phase (Sanhajariya et al., 2018). Further, for systemic toxins (such as α‐cbtx), a likely depot effect might result in a delay in the onset of their toxic effects, since the toxins first need to leave the bite site to enter the blood stream (Laustsen, 2019). Under such circumstances, where the toxins are released over time from the bite site into circulation, the antibodies would not be burdened with large amounts of toxins at once. Thus, we speculate that it would potentially be possible to neutralize the toxins using recycling antibodies at a lower dose and consequently at reduced cost, compared to using non‐recycling antibodies. However, to understand the overall effect of using recycling antibodies to neutralize toxins in a snakebite envenoming case requires further investigation. Nevertheless, the methodologies presented in this study could find broad applicability beyond snakebite envenoming as a general approach for the discovery of antibodies with pH‐dependent binding properties against potentially any target using in vitro display technologies.

## 4 MATERIALS AND METHODS

### 4.1 Purification of toxins

Venom from *Micrurus fulvius* was sourced as a composite from 67 individual specimens generously provided by Jack Facente of the “AGRITOXINS Venom Lab” in Florida, USA. Manually extracted and purified venom from *Micrurus diastema* was obtained from Instituto de Biotecnología, Universidad Nacional Autónoma de México (IBt‐UNAM) in Cuernavaca, Mexico. The primary phospholipase A_2_ (PLA_2_) neurotoxins from *M. fulvius* venom and a short‐chain α‐neurotoxin (SNTx) from *M. diastema* were isolated from the whole venoms by employing a C18 column based reversed‐phase high‐performance liquid chromatography (RP‐HPLC) methodology, as previously described (Vergara et al., 2014).

### 4.2 Biotinylation of antigen

Purified long‐chain α‐neurotoxins (LNTx), α‐cobratoxin (α‐cbtx) from *N. kaouthia* (Latoxan, France), and α‐bungarotoxin (α‐bgtx) from *B. multicinctus* (Latoxan, France) were dissolved in 1× phosphate‐buffered saline (PBS). The two LNTx (α‐cbtx and α‐bgtx) and the RP‐HPLC purified toxins PLA_2_ and SNTx were biotinylated using 1:1.25 (toxin: biotin reagent) molar ratio as previously described (Laustsen et al., 2018). The biotinylated toxins were purified using buffer exchange columns (Vivacon 500, Sartorius, 3000 Da molecular weight Cut‐Off) according to the manufacturer's protocol. Protein concentration was determined using the toxin's extinction coefficient and absorbance measurement with a NanoDrop One instrument. The degree of biotinylation was analyzed by MALDI‐TOF in an Ultraflex II TOF/TOF spectrometer (Bruker Daltonics).

### 4.3 Solution‐based phage display pH selection

The protocol for carrying out solution‐based phage display selections was adapted from previous work (Bonvin et al., 2015). The libraries used were the IONTAS naïve single‐chain variable fragment (scFv) phage display κ library (Schofield et al., 2007). Briefly, the phage display library was first blocked using 3% skimmed milk in PBS (MPBS) and then deselected using streptavidin‐coated beads (DynaBeads M280, Thermo Fisher #11205D). 100 nM biotinylated α‐cbtx was mixed with the deselected library and incubated for 1 h at pH 7.4. Phages bound to biotinylated α‐cbtx were then captured on streptavidin‐coated beads, and non‐specific phages were eliminated by washing thrice with PBS + 0.1% Tween (PBST), and twice with PBS. In the first round of phage display panning, all phages were eluted by trypsin digestion. In round two and round three, clones with pH‐dependent antigen binding properties were eluted by adding citrate buffer at pH 5.5 for 15–60 min or by trypsin. For the phages eluted using citrate buffer, trypsin was subsequently added to the eluted phages. The eluted phages were then used to infect TG1 cells as described before (Ledsgaard, Laustsen, et al., 2022).

### 4.4 Sub‐cloning and screening of scFvs

Sub‐cloning of scFv genes from phage outputs into the pSANG10‐3F vector and primary screening were performed as previously described (Laustsen et al., 2018). In short, *NcoI* and *NotI* restriction endonucleases sites were used to sub‐clone scFv genes from phagemids into the pSANG10‐3F vector, which was then transformed into *E. coli* strain BL21(DE3) (New England Biolabs). From each of the selection outputs, 184 colonies were picked, expressed in 96 well format, and assessed for binding to 50 nM of biotinylated α‐cobratoxin in an expression‐normalized capture (ENC) dissociation‐enhanced lanthanide fluorescence immunoassay (DELFIA) as described earlier, with a few modifications (Laustsen et al., 2018).

First, Nunc MaxiSorp plates (Invitrogen, 44‐2404‐21) were coated overnight with 50 μL of 2.5 μg/mL anti‐FLAG M2 antibody (Sigma Aldrich, F1804). Plates were washed thrice with PBS and blocked with 200 μL of 3% MPBS. Plates were washed thrice with PBS, and 60 μL of 0.5× unpurified scFv‐containing bacterial culture supernatant in 3% MPBS was added before incubating for 1 h at room temperature. Plates were washed thrice with PBS + 0.1% Tween and thrice with PBS before adding 50 μL of 50 nM biotinylated α‐cbtx in MPBS to each well. After 1 h of incubation, the plates were washed thrice with PBS + 0.1% Tween and thrice with PBS. Then, 1 μg/mL of Europium‐labeled Streptavidin (Perkin Elmer, 1244–360) in DELFIA Assay Buffer (Perkin Elmer, 4002–0010) was added. Following 30 min of incubation, plates were washed thrice with PBS + 0.1% Tween and thrice with PBS, and DELFIA Enhancement Solution (Perkin Elmer, 4001–0010) was added for detection of binding. Clones that gave a signal above 5,000 RFU were selected for further characterization.

### 4.5 ENC pH DELFIA and sequencing

To characterize the pH‐dependency of the α‐cbtx‐binding scFv candidates, a modified ENC DELFIA assay was performed. The assay was carried out as described above, with an additional pH‐elution step. The ENC pH DELFIA was performed in duplicates until after the incubation and washing of α‐cbtx, where 60 μL of citrate buffer at either pH 6.0 or pH 7.4 was added to each well. Following 60 min of incubation, plates were washed thrice with PBS + 0.1% Tween and thrice with PBS. Detection of biotinylated antigen was carried out as described in Section 4.4.

### 4.6 Bio‐layer interferometry (BLI) experiments

#### 4.6.1 Off‐rate screening of scFvs from bacterial culture supernatant

Prior to the assay, streptavidin (SA) biosensors were pre‐wetted for at least 10 min in 1× Kinetics Buffer (KB, ForteBio), followed by loading 1 μg/mL biotinylated α‐cbtx onto the SA biosensors. The screening was performed with a 120 s baseline step in 1× PBS pH 7.4. Thereafter, the toxin‐loaded biosensors were dipped in scFv‐containing bacterial supernatant wells for 300 s of association, followed by a dissociation step in 1x PBS pH 7.4 or pH 5.5 for 600 s. The tips were regenerated between each cycle in 10 mM Glycine pH 2.0 for 5 s followed by neutralization buffer (1× KB) for 5 s in total of 5 cycles. The experiment was performed at 25 °C with shaking at 1,000 rpm.

#### 4.6.2 Binding of Fab C08 to selected toxins

The binding of purified Fab C08 to biotinylated toxins LNTx, SNTx, and PLA_2_ was performed at pH 7.4 as described above, except that 300 nM of purified Fab C08 was used and 1× KB was used as the running buffer. The binding curves were analyzed using Octet® Analysis Studio 12.2.2.26 (ForteBio).

#### 4.6.3 Binding of Fab C08 to α‐cbtx in the presence of serum

To assess the binding of Fab C08 to α‐cbtx in the presence of human serum, 1 μg/mL biotinylated α‐cbtx was loaded on SA biosensor tips, followed by a 120 s baseline in 10% or 50% human serum (Sigma Aldrich) diluted in PBS. The tips were then dipped in wells containing 300 nM Fab C08 diluted in 10% or 50% human serum in PBS to allow association for 300 s and dissociation for 600 s. The binding curves were analyzed using Octet® Analysis Studio 12.2.2.26 (ForteBio).

#### 4.6.4 Determination of kinetic constants of Fabs

The off‐rates of purified Fabs at pH 7.4 and 5.5 were determined as described above except that the association was carried out in HEPES‐MES buffer at pH 7.4 containing C08 Fab from 18 to 300 nM or D11 Fab from 15 to 240 nM in a 2‐fold dilution, followed by dissociation in HEPES‐MES buffer at pH 7.4 or 5.5. Octet® Analysis Studio 12.2.2.26 (ForteBio) was used to fit the curves using 1:1 binding model with global fit to determine off‐rate constants at pH 7.4 and 5.5 and the binding affinities of the Fabs at pH 7.4.

#### 4.6.5 Binding of Fab C08 to recombinant α‐cbtx

The pH‐dependent antigen binding properties of Fab C08 to recombinantly expressed wildtype α‐cbtx and mutant α‐cbtx‐H18N was performed as described above, except that the loading was performed with 1 μg/mL biotinylated recombinant antigen and 300 nM of Fab C08 in HEPES‐MES buffer pH 7.4 was used for association. HEPES‐MES buffer at pH 7.4 or pH 5.5 was used for dissociation.

### 4.7 Nano differential scanning fluorimetry (NanoDSF) of Fabs

Melting temperature (T_M_) of the Fabs were determined using Nanotemper Prometheus Panta. Fabs were prepared at a concentration of 0.5 mg/mL in HEPES‐MES buffer at pH 7.4 or pH 5.5, loaded in capillaries (Standard ‐ NanoTemper Technologies), and subjected to a temperature gradient from 15 to 95 °C at a thermal ramping rate of 1 °C/min. The fluorescence emitted by the samples at 330 and 350 nm were measured to determine the T_M_. Data was processed using PR Panta Analysis v.1.4.3 software.

### 4.8 Antibody production

#### 4.8.1 Production of IgG1s

The reformatting of scFv into IgG1 was performed as previously described (Laustsen et al., 2018), except that the IgG1 expression vector for TPL0197_01_C08 contained the human kappa light chain instead of the human lambda light chain. ExpiCHO cells were cultured and transfected with expression vector according to the manufacturer's guidelines (Gibco™) following a protocol where ExpiFectamine™ CHO Enhancer and a single feed were added at day 1 and cells were maintained at 37 °C and 5% CO_2_ during the cultivation. The supernatant was collected at day 7 by removal of the cells through centrifugation at 300 g for 5 min, followed by an additional centrifugation at 1,000 *g* for 5 min. The supernatant was either used for purification on the same day or stored at −80 °C. Supernatant was thawed overnight at 4 °C, centrifuged, filtered, and loaded on a MabSelect column (Cytiva). 20 mM sodium phosphate and 150 mM NaCl (pH 7.2) was used for equilibration and washing of the column and elution was performed with 0.1 M sodium citrate (pH 3). Elution fractions were immediately neutralized by 1 M Tris (pH 9) using 1/5 volume of neutralization solution for 1 volume of elution fraction. Fractions of interest were pooled and loaded on a HiPrep 26/10 desalting column for buffer exchange to Dulbecco's PBS. Protein fractions were sterile‐filtered and concentrated by centrifugal filtration using an Amicon® Ultra‐15 centrifugal filter unit (30 kDa NMWL). The final concentration was determined by measuring the absorbance at 280 nm on a NanoDrop™ 2000 Spectrophotometer. Purity was checked by SDS‐PAGE. The purified protein was stored at 4 °C or −80 °C.

#### 4.8.2 Production of Fab

The reformatting of scFvs to Fabs and expression of Fabs was carried out as described in Section 4.8.1 except that expression vector of TPL0197_01_C08 contained the constant domain 1 sequence of the heavy chain and the kappa light chain, while that of 2554_01_D11 contained lambda light chain.

After expression, the collected supernatant was centrifuged and loaded on a 5‐mL HisTrap Excel column (Cytiva), equilibrated with 20 mM sodium phosphate (pH 7.4), 500 mM NaCl. The column was washed with 10 column volumes of 10 mM imidazole in 20 mM sodium phosphate (pH 7.4), 500 mM NaCl. Elution was performed in up‐flow mode with 20 column volumes of 500 mM imidazole in 20 mM sodium phosphate (pH 7.4), 500 mM NaCl. Protein containing fractions of interest were pooled and loaded on a HiPrep 26/10 desalting column for buffer exchange to Dulbecco's PBS. The protein was then concentrated by centrifugal filtration using an Amicon® Ultra‐15 centrifugal filter unit (3 kDa NMWL). The final concentration was determined by measuring the absorbance at 280 nm on a NanoDrop™ 2000 Spectrophotometer. Purity was checked by SDS‐PAGE. The purified protein was stored at 4 °C or −80 °C.

### 4.9 Production of α‐cbtx

#### 4.9.1 Plasmid construction for expression of recombinant wildtype α‐cbtx and mutant α‐cbtx‐H18N

*Komagataella phaffii* (formerly known as *P. pastoris*) codon optimized genes encoding 6xHis‐GSSG linker‐α‐cobratoxin (r‐α‐cbtx, Uniprot: P01391) and mutant 6xHis‐GSSG linker‐α‐cbtx‐H18N (α‐cbtx‐H18N) were obtained from Eurofins. For expression in *K. phaffii*, the toxin encoding genes and pPICZα A vector (Invitrogen) were restriction digested using EcoRI (FastDigest, Thermo Fisher) and XbaI (FastDigest, Thermo Fisher) restriction enzymes at 37 °C for 15 min, run on a 1% agarose gel, and gel extracted using Genejet Gel extraction kit as per the manufacturer's instructions. The restricted genes and vector were ligated using T4 DNA ligase (New England Biolabs) at room temperature for 20 min. 5 μL of ligation mixture was used to transform DH5α *E. coli* cells and the cells were plated in LB low salt (10 g/L Tryptone, 5 g/L Yeast Extract, 5 g/L NaCl) plates supplemented with 25 μg/mL Zeocin (ThermoFisher) and incubated at 37 °C overnight. The following day, single colonies from each of the plates were picked, inoculated in 5 mL LB low salt medium supplemented with 25 μg/mL zeocin, and cultured overnight at 37 °C and shaking at 220 rpm. The cultures were miniprepped using Genejet miniprep kit following the manufacturer's instructions and the obtained plasmids were sent for sequencing for verification. The sequence verified plasmids were selected for their expression in *K. phaffii* in the subsequent experiments.

#### 4.9.2 Expression of recombinant wildtype α‐cbtx and mutant α‐cbtx‐H18N in *K. phaffii*

10 μg of plasmid DNA of r‐α‐cbtx and α‐cbtx‐H18N were linearized using SacI (FastDigest, ThermoFisher) and then electroporated into the freshly prepared electrocompetent *K. phaffii* KM71H strain (ThermoFisher) using the Bio‐Rad Gene Pulser apparatus (Bio‐Rad, Hercules, CA, USA). Cells containing the integrated sequences were selected on YPDS plates (20 g/L Peptone, 10 g/L Yeast Extract, 100 mL/L Dextrose 20% (w/v), 182.2 g/L Sorbitol, 20 g/L Agar) supplemented with increasing concentrations of Zeocin (10, 100, or 1,000 μg/mL). Colonies from each r‐α‐cbtx and α‐cbtx‐H18N were picked from the 1,000 μg/mL plate, inoculated into separate 5 mL YPD medium tubes (20 g/L Peptone, 10 g/L Yeast Extract, 20% (w/v) Dextrose) and incubated overnight at 30 °C with shaking at 200 rpm. The following day, 1 mL of the saturated culture was transferred to 500 mL of buffered glycerol complex medium  medium (BMGY, 10 g/L Yeast Extract, 20 g/L Peptone, 0.1 M Potassium phosphate pH 6.0, 1.34% (w/v) YNB, 0.04 μg/mL Biotin, 1% (v/v) Glycerol) and grown for 24 h at 30 °C with shaking at 200 rpm. The culture was centrifuged at 5,000 g for 10 min, and the cell pellet was resuspended in 100 mL of buffered methanol complex medium (BMMY, 10 g/L Yeast Extract, 20 g/L Peptone, 0.1 M Potassium phosphate pH 6.0, 1.34% (w/v) YNB, 0.04 μg/mL Biotin, 0.5% (v/v) Methanol), whereafter the cells were grown at 25 °C for 4 days with the addition of methanol to a final concentration of 0.5% (v/v) every 24 h. After 96 h, the culture was centrifuged at 17,000 g for 30 min at 4 °C, the supernatant was collected, and filter‐sterilized by through a 0.2 μM membrane filter (Milipore). The filtered supernatant was then stored at 4 °C for subsequent purification steps.

#### 4.9.3 Purification of recombinant α‐cbtx

The filtered supernatants from *K. phaffii* were dialyzed 5 h and then overnight against 8 L of Wash Buffer (50 mM Sodium phosphate, pH 8.0, 20 mM imidazole) using 3 MWCO dialysis membranes (Snakeskin, ThermoFisher). The dialyzed media was subjected to His‐purification using gravity flow purification. First, 5 mL of equilibrated HIS‐Select® Nickel Affinity Gel resin (Sigma Aldrich) was washed and equilibrated with Wash Buffer. The resin was then mixed with the supernatant and incubated at 4 °C for 1 h with end‐over‐end rotation, following which the resin was transferred into chromatography columns, and the flow‐through fractions were collected. The columns were washed using 10 column volumes (CVs) of Wash Buffer followed by elution of the protein using 5 CVs of elution buffer (50 mM Sodium phosphate, pH 8.0, 400 mM imidazole). The eluted fractions were run on an SDS‐PAGE gel (NuPAGE™ 4%–12%, Bis‐Tris, ThermoFisher) where a low molecular weight protein ladder was also loaded (Thermo Scientific™ Spectra Multicolor Low Range Protein ladder). The fractions containing the eluted toxins were dialyzed twice using 3MWCO dialysis bags as described before against 1 L of PBS at 4 °C and subsequently concentrated using Amicon® Ultra‐15 Centrifugal Filters (3 kDa cut‐off, Millipore, Burlington, USA).

### 4.10 Circular dichroism (CD) spectroscopy of recombinant α‐cbtx

To assess the secondary structures of the recombinant α‐cbtx and α‐cbtx‐H18N, CD spectroscopy was performed following a previously described protocol (Rimbault et al., 2023). Briefly, the toxins were prepared at 0.5 mg/mL in PBS (pH 7.4). Far‐UV CD measurements were conducted using a JASCO J‐1500 spectrophotometer (Easton, MD, USA). The samples were loaded into a 0.1 mm quartz cuvette (Hellma) at 0.5 mg/mL. The spectrum was recorded by accumulating 10 measurements between 260 and 190 nm, with a bandwidth of 0.1 nm and intervals of 1 nm. The scan speed was set at 50 nm/s. The acquired spectra were processed using SpectraManager software (JASCO). Graphs depicting the CD spectra were generated using GraphPad Prism software (GraphPad Software). The ellipticity was transformed into molar ellipticity per residue considering the molecular weight of the recombinant proteins and the average weight per amino acid to enable normalized comparison across spectra.

### 4.11 pH‐dependent antigen binding properties evaluated by ELISA

96‐well EIA/RIA 3590 microplates (Corning) were coated with 100 μL of 0.5 μg/mL α‐cbtx (Latoxan, France) diluted in PBS overnight at 4 °C. The plates were blocked with 4% skimmed milk powder (M) (Sigma‐Aldrich) dissolved in PBS for 1 h, followed by washing four times with PBS containing 0.05% Tween 20 (PBS‐T) (Sigma‐Aldrich) (PBST). Unless stated otherwise, the following steps were carried out at pH 7.4 and 5.5, respectively, and the washing was conducted with PBST with the corresponding adjusted pH. Next, 100 μL of titrated amounts of 0.0009‐2 μg/mL for 2554_01_D11 and 0.003‐7.5 μg/mL for TPL0197_01_C08) of the IgG1s diluted in PBST‐M were added to the plates in a 2‐fold dilution and incubated at RT for 1 h. After washing, 100 μL of AP‐conjugated anti‐human IgG Fc (Sigma‐Aldrich) diluted 1:5000 in M‐PBST was added and incubated for 1 h. Thereafter, following washing with PBST, the bound proteins were detected by adding 100 μL of 1 mg/mL p‐nitropenylphosphate substrate tablets dissolved in diethanolamine buffer (pH 9.8) (Sigma‐Aldrich). The absorbance was measured at 405 nm using the Sunrise spectrophotometer (Tecan).

### 4.12 Molecular dynamics simulations

#### 4.12.1 Protonation dependent conformational ensembles

A previously published simulation protocol to capture CDR loop ensembles in solution was applied to characterize protonation dependent conformational changes of the C08 mAb free in solution(Fernández‐Quintero, Heiss, et al., 2020; Fernández‐Quintero, Kraml, et al., 2019; Fernández‐Quintero, Pomarici, et al., 2020). To predict the structure of the C08 antibody, the Antibody Modeler implemented in MOE with default settings was used. The two structurally close cysteine residues in the CDR‐H3 loop were bonded and the Fv structures for further molecular dynamics (MD) simulations in MOE were prepared (MOE, 2020; Labute, 2009).

#### 4.12.2 Constant pH simulations

To identify the most probable protonation states at pH 7.4 and pH 5.0, 100 ns of constant pH simulations were performed using the implementation for explicit solvent in the AMBER by Roitberg and coworkers (Swails et al., 2014). In this constant pH approach, the simulation is interrupted at periodic intervals, and protonation changes are attempted based on a Monte Carlo Metropolis criterion.

To neutralize the charges, the uniform background charge was used, which is required to compute long‐range electrostatic interactions (Darden et al., 1993). Using the tleap tool of the AmberTools20 (Case et al., 2020, p. 20) package, the structures were soaked in cubic water boxes of TIP3P water molecules with a minimum wall distance of 12 Å to the protein (El Hage et al., 2018; Gapsys & de Groot, 2019; Jorgensen et al., 1983). For all simulations, parameters of the AMBER force field 14SB were used (Maier et al., 2015).

#### 4.12.3 Enhanced sampling and classical MD simulations

To enhance the sampling of the conformational space, well‐tempered bias‐exchange metadynamics (Barducci et al., 2008; Domene et al., 2015) simulations were performed in GROMACS (Abraham et al., 2015; Pronk et al., 2013) with the PLUMED 2 implementation (Tribello et al., 2014, p. 2). Metadynamics was chosen as simulation approach since it enhances sampling on predefined collective variables (CV). The sampling was accelerated by a history‐dependent bias potential, which is constructed in the space of the CVs. As collective variables, a well‐established protocol, boosting a linear combination of sine and cosine of the ψ torsion angles of all six CDR loops calculated with functions MATHEVAL and COMBINE implemented in PLUMED 2, was used (Tribello et al., 2014, p. 2). As discussed previously, the ψ torsion angle captures conformational transitions comprehensively (Ramachandran et al., 1963). The underlying method presented here has been validated in various studies against experimental results (Fernández‐Quintero, Loeffler, et al., 2019; Fernández‐Quintero, Loeffler, et al., 2020; Fernández‐Quintero, Pomarici, et al., 2020). A Gaussian height of 10.0 kJ/mol and a width of 0.3 rad was employed. Gaussian deposition occurred every 1,000 steps, and a biasfactor of 10 was used. 500 ns of bias‐exchange metadynamics simulations were performed for the prepared Fv structures. The resulting trajectories were aligned to the whole Fv and clustered with cpptraj (Roe & Cheatham, 2013) using the average linkage hierarchical clustering algorithm with a RMSD cut‐off criterion of 1.2 Å resulting in a large number of clusters. The cluster representatives for the antibody fragments were equilibrated and simulated for 100 ns using the AMBER 20 simulation package (Case et al., 2020).

Molecular dynamics simulations were performed in an NpT ensemble using pmemd.cuda (Salomon‐Ferrer et al., 2013). Bonds involving hydrogen atoms were restrained by applying the SHAKE algorithm (Miyamoto & Kollman, 1992), allowing a time step of 2 fs. Atmospheric pressure of the system was preserved by weak coupling to an external bath using the Berendsen algorithm (Berendsen et al., 1984). The Langevin thermostat was used to maintain the temperature during simulations at 300 K (Adelman & Doll, 1976; Doll et al., 1975). In total, 94.4 μs of simulation time for the C08 antibody at pH 7.4 and pH 5.0 were accumulated.

#### 4.12.4 Analysis and visualization—Conformational state characterization

With the obtained trajectories, a time‐lagged independent component analysis (tICA) using the python library PyEMMA 2 employing a lag time of 10 ns was performed. tICA was applied to identify the slowest movements of the investigated Fv fragments and consequently to obtain a kinetic discretization of the sampled conformational space (Bowman et al., 2014; Chodera & Noé, 2014; Scherer et al., 2015, p. 2). tICA is a dimensionality reduction technique that detects the slowest‐relaxing degrees of freedom and facilitates kinetic clustering, which is a crucial pre‐requisite for building a Markov‐state model (Pérez‐Hernández & Noé, 2016). It linearly transforms a set of high‐dimensional input coordinates to a set of output coordinates by finding a subspace of “*good reaction coordinates*.” Thereby, tICA finds coordinates of maximal autocorrelation at a given lag time. The lag time sets a lower limit to the timescales considered in the tICA and the Markov‐state model. Accordingly, tIC1 and tIC2 represent the two slowest degrees of freedom of the systems. Based on the tICA conformational spaces, thermodynamics and kinetics were calculated with a Markov‐state model (MSM) (Chodera & Noé, 2014) using PyEMMA 2, which employs the k‐means clustering algorithm to define microstates and the PCCA+ clustering algorithm (Röblitz & Weber, 2013) to coarse‐grain the microstates to macrostates. MSMs are network models which provide valuable insights for conformational states and transition probabilities between them, as they allow for the identification of boundaries between two states (Bowman et al., 2014; Chodera & Noé, 2014). Basically, MSMs coarse‐grain the dynamics of the system, which reflect the free energy surface and ultimately determine the structure and dynamics of the system. Thus, MSMs provide important insights and enhance the understanding of states and transition probabilities, facilitating a quantitative connection with experimental data (Karush, 1961). To build the MSM, the backbone torsions of the respective CDR loops were used, 250 microstates using the k‐means clustering algorithm were defined, and a lag time of 10 ns was applied.

Furthermore, for each CDR loop state, we computed the relative interdomain orientations between the antibody variable domains (V_H_ and V_L_) using six measurements (five angles and a distance) using ABangle (Dunbar et al., 2013; Fernández‐Quintero, Hoerschinger, et al., 2020). The ABangle script can calculate these measures for an arbitrary Fv region by aligning the consensus structures to reference coordinate set positions and fitting the planes and distance vector from this alignment. This available online tool was combined with an in‐house python script to reduce computational effort and to visualize our simulation data over time (Millman & Aivazis, 2011). The in‐house script makes use of ANARCI (Dunbar & Deane, 2016) for fast local annotation of the variable fragment (Fv) region and pytraj from the AmberTools package (David et al., 2016) for rapid trajectory processing. Furthermore, we used PyMOL to visualize the antibody structure (Schrodinger, 2015).

## AUTHOR CONTRIBUTIONS

**Tulika Tulika:** Conceptualization; investigation; writing – original draft; methodology; validation; writing – review and editing; data curation; visualization; software; supervision. **Rasmus W. Pedersen:** Conceptualization; investigation; writing – original draft; methodology; validation; visualization; data curation; writing – review and editing. **Charlotte Rimbault:** Conceptualization; methodology; supervision; writing – review and editing. **Shirin Ahmadi:** Writing – review and editing; supervision; conceptualization. **Line Ledsgaard:** Supervision; writing – review and editing; conceptualization; methodology. **Markus‐Frederik Bohn:** Writing – review and editing; project administration; supervision; resources; investigation; software. **Anne Ljungars:** Supervision; project administration; writing – review and editing. **Bjørn G. Voldborg:** Resources; writing – review and editing; project administration. **Fulgencio Ruso‐Julve:** Methodology; writing – review and editing. **Jan Terje Andersen:** Writing – review and editing; resources. **Andreas H. Laustsen:** Conceptualization; supervision; funding acquisition; writing – review and editing; project administration. **Esperanza Rivera‐de‐Torre:** Methodology; investigation; writing – review and editing; supervision. **Monica L. Fernández‐Quintero:** Methodology; writing – original draft; writing – review and editing; investigation; conceptualization; data curation. **Johannes R. Loeffler:** Methodology; formal analysis.

## CONFLICT OF INTEREST STATEMENT

The authors declare no conflicts of interest.

## Supporting information


**Figure S1:** Bio‐layer interferometry (BLI) binding curves of Fab C08 and toxins (A) Binding of 300 nM Fab C08 to α‐cobratoxin (α‐cbtx, Uniprot ID: P01391), α‐bungarotxin (Uniprot ID: P60615) which is a long‐chain α‐neurotoxin (LNTx) structurally similar to α‐cbtx, a short‐chain α‐neurotoxin (SNTx), and a phospholipase A_2_ toxin (PLA_2_). (B) Binding of 300 nM Fab C08 to α‐cbtx in the presence of human serum diluted in PBS to 10% and 50% (v/v).

**Figure S2:** SDS‐PAGE of recombinant α‐cobratoxins (α‐cbtx) and their Circular Dichroism (CD) spectra. (A) SDS‐PAGE (NuPAGE™ 4%–12%, Bis‐Tris, ThermoFisher) showing the migration of the native α‐cbtx, recombinant wildtype α‐cbtx (r‐α‐cbtx), and mutant α‐cbtx‐H18N. All the samples were boiled for 15 min at 95 °C in the presence of 1 mM DTT to ensure the complete denaturation of the protein and reduction of the disulfide bonds. A protein ladder is also present on the gel, molecular weights are indicated in kDa at the left side of image. (B) Far‐UV CD spectra of the r‐α‐cbtx (shown in black) and α‐cbtx‐H18N (shown in red).
