# Supplementary figures and images for: Phage display assisted discovery of a pH‐dependent anti‐α‐cobratoxin antibody from a natural variable domain library
Source: Protein Sci. 2023 Dec 1;32(12):e4821. doi: 10.1002/pro.4821 (PMC10659949; doi:10.1002/pro.4821)

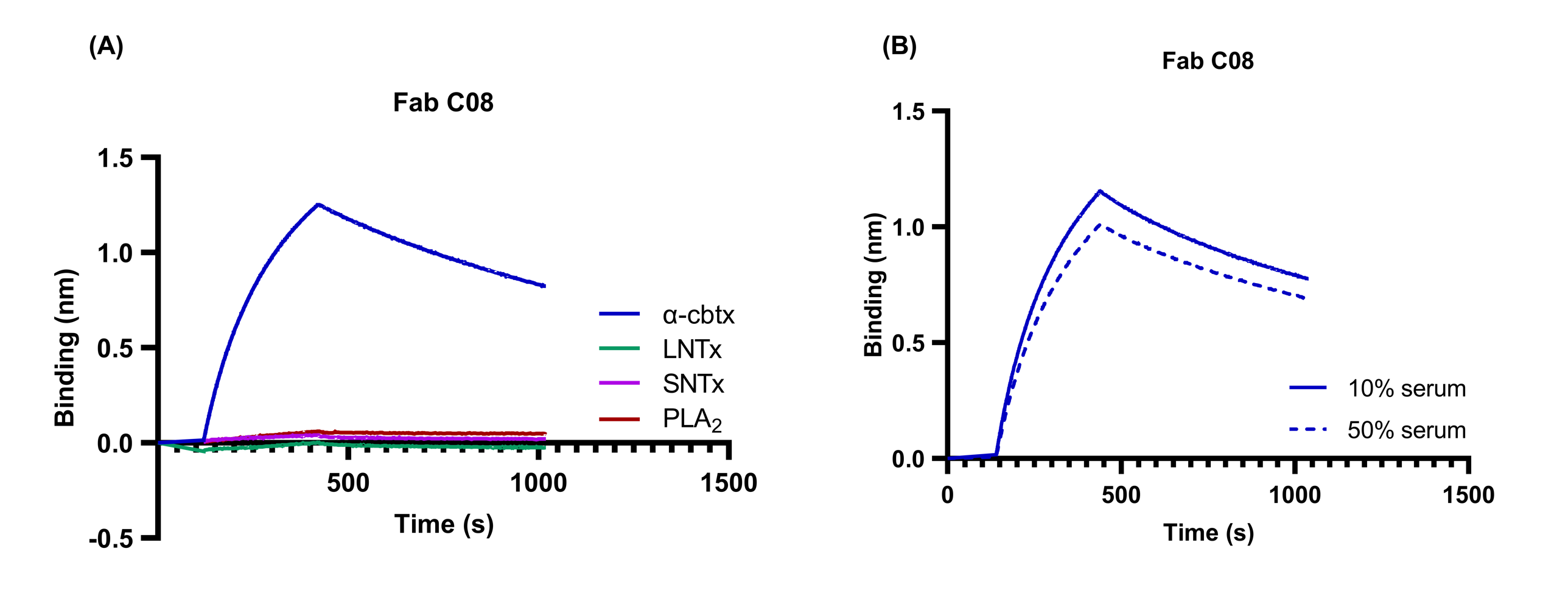

Supplement: Supplementary file 1 — Figure S1: Bio‐layer interferometry (BLI) binding curves of Fab C08 and toxins (A) Binding of 300 nM Fab C08 to α‐cobratoxin (α‐cbtx, Uniprot ID: P01391), α‐bungarotxin (Uniprot ID: P60615) which is a long‐chain α‐neurotoxin (LNTx) structurally similar to α‐cbtx, a short‐chain α‐neurotoxin (SNTx), and a phospholipase A2 toxin (PLA2). (B) Binding of 300 nM Fab C08 to α‐cbtx in the presence of human serum diluted in PBS to 10% and 50% (v/v). [file PRO-32-e4821-s002.zip › Supplementary-figure-1_1.tif]
